# Case report: Double-chambered right ventricle diagnosed in a middle-aged female with hypertrophic cardiomyopathy and atrial flutter: A rare case

**DOI:** 10.3389/fcvm.2022.937758

**Published:** 2022-07-22

**Authors:** Junye Ge, Tong Hu, Yan Liu, Qian Wang, Guanqi Fan, Chuanzhen Liu, Jun Zhang, Shiming Chen, Kellina Maduray, Yun Zhang, Tongshuai Chen, Jingquan Zhong

**Affiliations:** ^1^The Key Laboratory of Cardiovascular Remodeling and Function Research, Chinese Ministry of Education, Chinese National Health Commission and Chinese Academy of Medical Sciences, The State and Shandong Province Joint Key Laboratory of Translational Cardiovascular Medicine, Department of Cardiology, Qilu Hospital, Cheeloo College of Medicine, Shandong University, Jinan, China; ^2^Department of Radiology, Qilu Hospital, Shandong University, Jinan, China; ^3^Department of Cardiovascular Surgery, Qilu Hospital, Shandong University, Jinan, China; ^4^Department of Pathology, Qilu Hospital, Shandong University, Jinan, China; ^5^Department of Cardiology, Qilu Hospital (Qingdao), Cheeloo College of Medicine, Shandong University, Qingdao, China

**Keywords:** double-chambered right ventricle, hypertrophic cardiomyopathy, atrial flutter, echocardiography, cardiac magnetic resonance

## Abstract

Double-chambered right ventricle (DCRV) is a rare congenital heart defect in adults, manifesting with progressive right ventricular outflow tract obstruction. We describe the first case of DCRV coexisting with hypertrophic cardiomyopathy, which is complicated by atrial flutter. A middle-aged woman with recurrent symptomatic atrial flutter who had previously been diagnosed with biventricular hypertrophic cardiomyopathy was admitted to our department. Echocardiography and cardiac magnetic resonance revealed asymmetrical interventricular septal hypertrophy, and abnormal muscle bundles within the right ventricle, generating an obstructive gradient. Genetic testing detected a hypertrophic cardiomyopathy-associated mutation: *MYH7*, c.4135G > A, p. Ala1379Thr. A diagnosis of DCRV complicated by hypertrophic cardiomyopathy and atrial flutter was made. Surgical intervention was performed, which included radiofrequency ablation, removal of abnormal muscle bundles, and ventricular septal defect repair. Intraoperative transesophageal echocardiography demonstrated the well-corrected right ventricular outflow tract. Free of early postoperative complications, the patient was discharged in sinus rhythm on the 11th day after the surgery. Unfortunately, the patient died from a sudden death 38 days following the surgery. In conclusion, the coexistence of DCRV with hypertrophic cardiomyopathy in patients is an uncommon condition. The present case highlights the importance of diagnostic imaging in the management of this disorder.

## Introduction

Double-chambered right ventricle (DCRV) is a rare congenital heart disease (CHD) characterized by abnormal muscle bundles, resulting in intra-cavity obstruction in which the right ventricle (RV) is divided into a high-pressure chamber near the tricuspid valve and a low-pressure chamber near the pulmonary valve (PV) (1). DCRV accounts for only 0.5–2% of all CHD cases (2). Hypertrophic cardiomyopathy (HCM) is the most common genetic cardiac disease. About 2/3 of patients experience left ventricular outflow tract obstruction, known as hypertrophic obstructive cardiomyopathy (HOCM) (3). Yamamoto et al. first described HOCM in a DCRV patient with Noonan syndrome (2). Imaging examinations play an important role in the diagnosis of DCRV with HCM. Here, we present a case of DCRV complicated by HCM and atrial flutter (AFL). To our knowledge, such a combination has never been reported before.

## Case presentation

**FIGURE 1:**
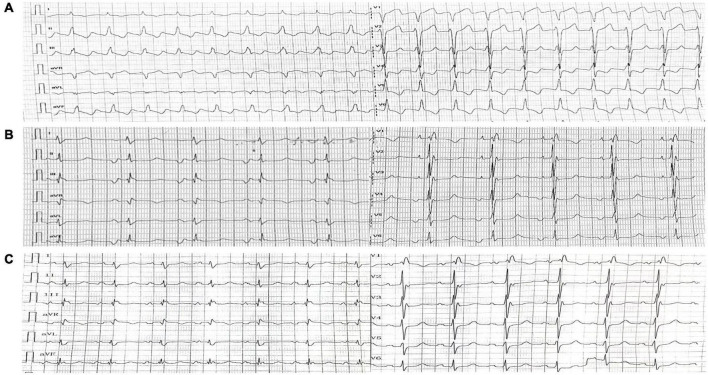
Electrocardiography. **(A)** Electrocardiography upon admission showed Type I atrial flutter. **(B)** Post-operative electrocardiography (1st day) showed coronary sinus rhythm. **(C)** Post-operative electrocardiography (8th day) showed recovered sinus rhythm.

**FIGURE 2:**
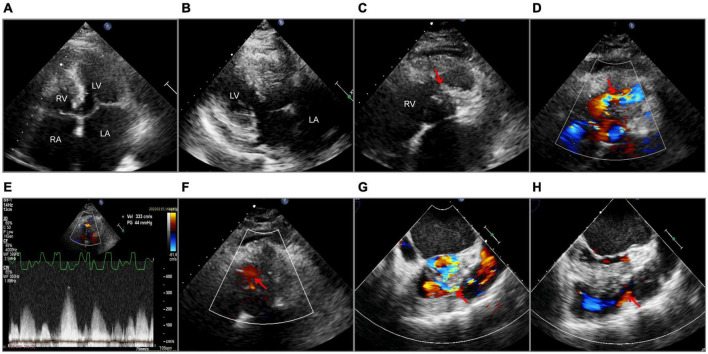
Echocardiography. Transthoracic echocardiography **(A–F)**. **(A)** A 2-D apical four-chamber view with enlarged atria. **(B)** Left ventricular hypertrophy and interventricular septal hypertrophy. **(C)** Muscular septation within the right ventricle (the red arrow). **(D)** Flow acceleration showed by Color Doppler at the right ventricular outflow tract (the red arrow). **(E)** The systolic pressure gradient of the right ventricular outflow tract. **(F)** Ventricular septal defect (the red arrow) detected by Color Doppler. Transesophageal echocardiography **(G,H)**. Color Doppler flow of the right ventricular outflow tract before **(G)** and after operation **(H)**, as shown by the red arrows. LA, left atrium; RA, right atrium; LV, left ventricle; RV, right ventricle; Vel, velocity; PG, pressure gradient.

**FIGURE 3:**
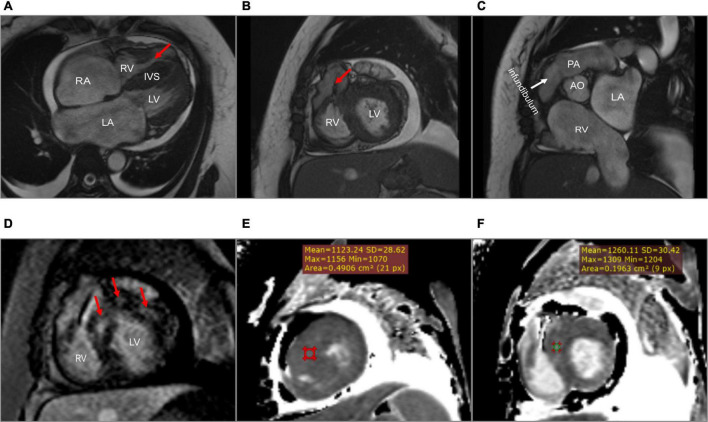
Cardiac magnetic resonance. **(A)** A dumbbell-shaped right ventricle indicated right ventricular outflow tract obstruction (the red arrow). **(B)** A flow void sign at the right ventricular outflow tract (the red arrow). **(C)** No stenosis of infundibulum, pulmonary valve, and pulmonary artery. **(D)** Scattered patchy late gadolinium enhancement within the left ventricular myocardium (red arrows), particularly in the left ventricular anterior wall and the interventricular septum. **(E,F)** The native T1 mapping revealed the markedly increased T1 value at the basal interventricular septum from 1,123 to 1,260 ms. LA, left atrium; RA, right atrium; LV, left ventricle; RV, right ventricle; IVS, interventricular septum; PA, pulmonary artery; AO, aorta.

A 45-year-old woman presenting with a 10-year history of palpitations and recent aggravation accompanied with dyspnea was referred to our center. She was originally diagnosed with biventricular HCM yet did not receive relevant treatment. Assessment of family medical history revealed that her mother died of a sudden cardiac death (SCD). Upon physical examination, the patient’s vital signs were stable, with the exception of tachycardia (112 beats/min) and a loud grade 4/6 systolic ejection murmur in the 4th intercostal space along the left sternal border. Routine 12-lead electrocardiography displayed recurrent AFL at a ventricular rate of 112 beats/min (Figure 1A). Transthoracic echocardiography (TTE) revealed mildly reduced left ventricular ejection fraction (EF: 45%), mild pericardial effusion, biatrial enlargement (left atrium: 54 mm × 68 mm × 58 mm; right atrium: 59 mm × 43 mm) (Figure 2A), biventricular hypertrophy and asymmetrical interventricular septal hypertrophy (20 mm) (Figure 2B). The anomalous muscle bundles inferior to the infundibulum divided the RV into two cavities, inducing right ventricular outflow tract (RVOT) obstruction (Figures 2C,D and Supplementary Videos 1, 2). The peak pressure gradient of RVOT was approximately 44 mmHg, and the forward flow velocity was measured at 333 cm/s (Figure 2E). The obstructive gradient, however, may have been underestimated due to the inability of color Doppler to align turbulent flow. Furthermore, a small left to right membranous ventricular septal defect (VSD) (0.3 cm) shunt was discovered in the high-pressure chamber (Figure 2F and Supplementary Video 3). Cardiac magnetic resonance (CMR) further confirmed the presence of sub-infundibular obstruction, biventricular hypertrophy, and interventricular septal hypertrophy (basal segment: 22.5 mm, middle segment: 25.5 mm, distal segment: 21 mm). A dumbbell-shaped RV and flow acceleration at the RVOT were observed during systole (Figures 3A,B and Supplementary Video 4). CMR assessed RV function with a right ventricular ejection fraction of 63%, right ventricular end-diastolic volume of 61 ml, and right ventricular end-systolic volume of 23 ml. No signs of stenosis were identified in the infundibulum, PV, and pulmonary artery (PA) (Figure 3C). Scattered patchy late gadolinium enhancement (LGE) was seen within the left ventricular myocardium, particularly in the left ventricular anterior wall and interventricular septum (Figure 3D). The native T1 mapping revealed a markedly increased T1 value at basal IVS from 1,123 to 1,260 ms (Figures 3E,F). The reference native T1 value of our 1.5T scanner was 1,035 ms. Genetic testing detected one HCM-associated mutation: *MYH7*, c.4135G > A, p. Ala1379Thr. *MYH7* is the most frequent pathogenic gene of HCM, accounting for 40–44% of HCM cases (3).

**FIGURE 4:**
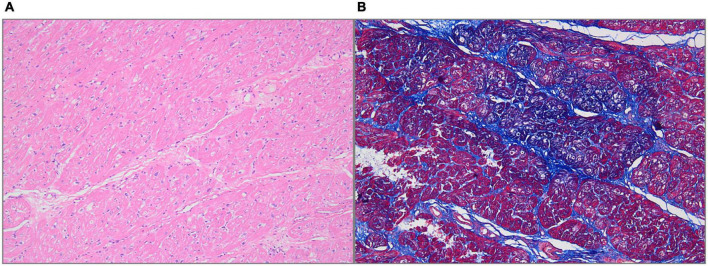
Histologic features. **(A)** Hematoxylin-Eosin staining showed myocardial fiber hypertrophy, degeneration and necrosis of some myocardial fibers, and hyaline degeneration (100× magnification). **(B)** Masson staining showed fibrous tissue hyperplasia (100× magnification).

The final diagnosis was DCRV complicated by HCM and AFL. Despite the administration of an adequate amount of amiodarone, successful conversion of AFL was not achieved. Since the patient suffered from renal dysfunction (serum creatinine: 175 μmol/L; GFR by Cockcroft-Gault: 39.49 ml/min), oral rivaroxaban (15 mg) was administered daily following admission. The HCM Risk-SCD formula was applied for risk stratification, resulting in a 4.26% 5-year risk of SCD. The decision not to implant an ICD was made after carefully analyzing the clinical benefits and the patient’s preferences. Surgery was performed via the median sternotomy approach. AFL was terminated by ablation of the cavotricuspid isthmus. Intraoperatively, a 1.5-cm subvalvular VSD was identified, larger than that shown on TTE. The muscular stenosis tunnel was resected, and a pericardial patch was used for VSD repair. Intraoperative transesophageal echocardiography (TEE) demonstrated well-corrected RVOT (Figures 2G,H and Supplementary Videos 5–6). The pathology report revealed myocardial fiber hypertrophy, degeneration and necrosis of some myocardial fibers, fibrous scar formation, local subendocardium fibrous tissue hyperplasia, hyaline degeneration, and hyperplasia of elastic fibers in the focal subendocardium, all of which were consistent with HCM (Figure 4). On the first postoperative day, the electrocardiography showed coronary sinus rhythm (Figure 1B). Sinus rhythm recovered on the 8th day after the operation (Figure 1C). The patient was discharged on the 11th day after the surgery with no early postoperative complications. Unfortunately, the patient died from a sudden death 38 days after the surgery. Since no autopsy was performed, the cause of death could not be confirmed. The mechanism of death was presumed to be arrhythmic.

## Discussion

Double-chambered right ventricle is a rare disease in which the pressure gradient across the obstruction frequently surpasses 20 mmHg, accompanied by a high pulmonary blood flow volume (4). Approximately, 80–90% of patients additionally present with other cardiac anomalies, including VSD (seen in up to 90% of patients with DCRV), atrial septal defect, subaortic stenosis, aortic valve regurgitation, PV stenosis, and so on (4). However, DCRV complicated by HCM is an extremely rare coexistence. Several cases reported this unique condition complicated with HCM (2, 5, 6). With respect to the combination of arrhythmias, Alvarez et al. reported that, in two adult patients diagnosed with DCRV, complicated by sustained monomorphic ventricular tachycardia, tachycardia did not reoccur after surgical excision of the abnormal muscle bundles (7). This report is the first to describe DCRV complicated by HCM and AFL; therefore, it offers guidance for clinical practice.

The symptoms of DCRV are often atypical, similar to those that present in other types of cardiovascular diseases. The most prevalent symptoms are shortness of breath and reduced exercise endurance (8). As the severity of RV blockage increases in adults, even light physical exertion might cause considerable dyspnea. DCRV is most commonly diagnosed in infants and children. However, it can occasionally be detected in adults due to misdiagnosis or the absence of symptoms at a young age (8, 9). There is, currently, a knowledge gap regarding DCRV diagnosis, which is further exacerbated by HCM. In our case, the patient has been previously diagnosed with HCM and AFL, while DCRV was overlooked. According to Said et al., preoperative TTE had a 74% success rate in detecting DCRV; however, as knowledge of DCRV grew, the accuracy of diagnosis improved (6). Thus, considerable experience and advanced diagnostic techniques are of vital importance. Echocardiography is an effective diagnostic tool for DCRV. TTE is initially used to assess cardiac structure; however, limitations arise when diagnosing DCRV in adults due to the unique shape of the RV and its retrosternal position (10). Obesity and emphysema are additional factors that limit the use of TTE in adults (11). The subcostal view tends to provide better visualization of the RVOT, while it is much clearer in infants or children than in adults (10). TTE successfully confirmed the presence of DCRV complicated by HCM and VSD in our case. Due to improved visualization of the RVOT and the ability to precisely observe aberrant muscle bundles and estimate the gradient pressure, TEE is a more effective method for diagnosing DCRV (10). Hoffman et al. compared the success rates of the two echocardiography methods in the diagnosis of DCRV; the study found that TEE was more effective, and the combined application of both methods further improved the accuracy (4). At present, CMR is the best non-invasive examination method for the diagnosis of DCRV, while TEE is deemed unnecessary (12). By carefully modifying the direction and angle of CMR scans, the features of the RVOT and the anatomy of the heart can be clearly shown. Comparatively, TEE is confined to the interface between the esophagus and the heart. In addition, LGE by CMR is able to detect the presence of multiple myocardial fibrosis. If VSD is opened in the proximal high-pressure cavity, the shunt velocity from left to right is low, limiting the shunt volume. Therefore, VSD was undetectable by CMR in our case. Cardiac catheterization and angiography are invasive means often used to confirm the diagnosis and obtain precise blood flow information (12). However, with the continuous improvement of non-invasive techniques, such as echocardiography and CMR, angiography may gradually lose position when diagnosing DCRV (8).

As the only RVOT lesion inferior to the infundibulum (1), DCRV needs to be distinguished from other types of lesions that produce RVOT obstruction, such as anomalies at the infundibular region, PV, the superior valvular region, left or right PA, and peripheral PA (12, 13). The position of the auscultation murmur indicated a lower level of obstruction in this patient. We confirmed the diagnosis of DCRV by echocardiography and CMR without abnormalities in the infundibular region, PV, and PA. It is important to note that the diagnosis of DCRV coexisting with VSD is, to some extent, similar to tetralogy of Fallot (TOF). DCRV has a pressure difference in the RV between the proximal high-pressure cavity and the distal low-pressure cavity, whereas the infundibular region is generally normal. However, for TOF, a pressure difference exists between the PA and RV or within the transition zone in the infundibular region due to PV or infundibulum stenosis.

Although DCRV anatomy is congenital and the RVOT may be non-obstructive at birth, the progressive hypertrophy of aberrant muscle bundles causes RVOT obstruction to develop or worsen (8, 14). RVOT obstruction progresses rapidly in young adults with DCRV, who usually present with severe obstruction around the age of 30 or 40 (15). Nonetheless, it was discovered that, in some patients who did not receive surgical treatment, the severity of RVOT blockage did not worsen over time (16). Surgical treatment is considered to be an effective way to remove RVOT obstruction. The severity of the blockage and lesion usually determines whether or not surgery is necessary (4). Since some patients with DCRV do not experience progressive RVOT blockage during the normal course of the disease, basic follow-up is sufficient for patients with no significant lesions and little or no pressure gradient in the RV (4, 16, 17). Negative inotropic drugs, such as beta-blockers and calcium channel antagonists, may be effective in improving symptoms (2, 12). If the obstruction worsens during follow-up, surgical repair may be considered. McElhinney et al. recommend surgical repair in adults who present with symptoms or severe obstruction (pressure gradient more than 40 mmhg) despite not having any symptoms (17). Some authors advocated for early surgical therapy even in the absence of symptoms, given the progressive development of blockage and symptoms (8). The European Society of Cardiology guideline recommends surgical treatment of DCRV, even if the obstruction pressure gradient is low (IIa) (13). Further evidence is expected to be gathered to determine the optimum surgical option. With a low recurrence and mortality rate, the long-term prognosis of surgical treatment is excellent, resulting in most patients remaining symptom free after surgery (8). In our case, the morphology of ECG changed significantly on the 1st day after operation: P wave was deeply inverted (negative) in leads II, III, and aVF, and positive in lead V1. According to its characteristics, the P wave was indicative of coronary sinus rhythm. Coronary sinus rhythm might be related to the delayed recovery of sinus node function after the conversion of AFL. The ECG showed sinus P wave morphology on the 8th day after the operation. The P wave was wide with a notch on the peak caused by bi-atrial enlargement. A complete right bundle branch block produced pathological ST segments in leads V1-V3, which could be related to surgical excision.

The combination of HCM is one of the unique features of this case. A study showed that the annual incidence of cardiovascular mortality is 1–2% in adult patients with HCM (18). SCD is one of the leading causes of death in patients with HCM, which is often correlated with lethal arrhythmias, including ventricular tachycardia, ventricular fibrillation, and a complete atrioventricular conduction block. Regarding HCM management in clinical practice, the risk stratification of SCD is of the utmost importance. ICD implantation is the only reliable way to prevent SCD in patients with HCM. Predictive factors of SCD in adult patients with HCM include early age of the onset, non-sustained ventricular tachycardia, maximum LV wall thickness of ≥ 30 mm, family history of early SCD, unexplained syncope, enlarged left atrial size, severe left ventricular outflow tract obstruction, LGE, multiple genetic mutations, and so on (19). In this case, the HCM Risk-SCD model was used to stratify risk. It divides adult patients with HCM into three risk levels: low risk (5-year risk < 4%), intermediate risk (5-year risk with ≥ 4%– < 6%), high risk (5-year risk ≥ 6%) (19). ICD implantation is suggested in high-risk patients and generally not recommended in low-risk patients. In intermediate-risk patients, ICD may be taken into consideration. In our case, after discussion and shared decision-making, ICD implantation was not performed.

There were some limitations in this case. Sudden death occurred 38 days after the surgery. Thromboembolic events are one of the most common complications of AFL or atrial fibrillation with biatrial enlargement. In this patient, adequate rivaroxaban was given after admission and discharge. Moreover, preoperative TEE showed no thrombus in the left atrium. Therefore, it may be possible to rule out the occurrence of thromboembolic events. CMR showed scattered patchy LGE in the left ventricular myocardium. In a large cohort study, myocardial fibrosis revealed by CMR was linked to an increased risk of ventricular tachyarrhythmias in patients with HCM (20). Since no autopsy was performed, it was impossible to ascertain the exact cause of death. We postulated that ventricular arrhythmia, which may be related to multiple myocardial fibrosis, was a significant contributing factor.

In conclusion, DCRV is typically accompanied with other lesions and rarely manifests as a standalone abnormality. Echocardiography and CMR are essential in the diagnosis of DCRV. Due to the low risk of complications and a favorable long-term prognosis, surgery has been proved to be fundamental in removing the obstruction. In patients with HCM, with a moderate 5-year SCD risk determined by the HCM Risk-SCD model, LGE may be indicative of a poor prognosis. Clinicians should recognize early the risk factors of SCD and stratify risk to make the best decision in patients with DCRV complicated by HCM.

## Data availability statement

The raw data supporting the conclusions of this article will be made available by the authors, without undue reservation.

## Ethics statement

Written informed consent was obtained from the participant/s for the publication of this case report. Written informed consent was obtained from the individual(s) for the publication of any potentially identifiable images or data included in this article.

## Author contributions

JG, TH, YL, and TC contributed to conception and design of the case. JG and TH wrote the first draft of the manuscript. QW, GF, CL, JZ, SC, KM, and YZ wrote sections of the manuscript. YL, QW, CL, JZ, SC, and YZ contributed to the clinical treatment of this case. TC and JQZ contributed to the review of the manuscript. All authors contributed to manuscript revision, read, and approved the submitted version.

## Conflict of interest

The authors declare that the research was conducted in the absence of any commercial or financial relationships that could be construed as a potential conflict of interest.

## Publisher’s note

All claims expressed in this article are solely those of the authors and do not necessarily represent those of their affiliated organizations, or those of the publisher, the editors and the reviewers. Any product that may be evaluated in this article, or claim that may be made by its manufacturer, is not guaranteed or endorsed by the publisher.

## Supplementary material

Supplementary Video 1 Transthoracic echocardiography showed anomalous muscle bundles at the right ventricular outflow tract.

Supplementary Video 2 Transthoracic echocardiography showed flow acceleration at the right ventricular outflow tract.

Supplementary Video 3 Transthoracic echocardiography showed ventricular septal defect.

Supplementary Video 4 Cardiac magnetic resonance clearly showed the double-chambered right ventricle and accelerated blood flow during systole.

Supplementary Video 5 Transesophageal echocardiography showed flow acceleration at the right ventricular outflow tract.

Supplementary Video 6 Transesophageal echocardiography showed a well-corrected right ventricular outflow tract.

## Abbreviations

DCRV: double-chambered right ventricle
RVOT: right ventricular outflow tract
CHD: congenital heart disease
RV: right ventricle
HCM: hypertrophic cardiomyopathy
HOCM: hypertrophic obstructive cardiomyopathy
AFL: atrial flutter
SCD: sudden cardiac death
TTE: transthoracic echocardiography
EF: ejection fraction
VSD: ventricular septal defect
CMR: cardiac magnetic resonance
LGE: late gadolinium enhancement
PV: pulmonary valve
PA: pulmonary artery
TEE: transesophageal echocardiography.
